# Supplementary material for: Nature’s Role in Supporting Health during the COVID-19 Pandemic: A Geospatial and Socioecological Study
Source: Int J Environ Res Public Health. 2021 Feb 24;18(5):2227. doi: 10.3390/ijerph18052227 (PMC7967714; doi:10.3390/ijerph18052227)
Supplement: Supplementary file 1 [file ijerph-18-02227-s001.pdf]

Supplementary Materials

Link S1: Research questionnaire preview

Table S1. Association between NDVI, forest presence and abundance, and self-reported mental wellbeing during the COVID-19 pandemic.

	Forest Pr 50 m	Forest Ab 50 m	Forest Pr 100 m	Forest Ab 100 m	Forest Pr 250 m	Forest Ab 250 m	Forest Pr 500 m	Forest Ab 500 m
Model 1: Unadjusted	0.78 (0.39, 1.54) *p* = 0.46	0.81 (0.48, 1.37) *p* = 0.40	0.87 (0.53, 1.42) *p* = 0.57	0.82 (0.59, 1.14) *p* = 0.21	1.26 (0.8, 1.99) p = 0.32	1.02 (0.91, 1.15) *p* = 0.68	1.41 (0.6, 3.34) *p* = 0.42	1.02 (0.91, 1.15) *p* = 0.68
Model 2: Adjusted for age	0.72 (0.36, 1.45) *p* = 0.36	0.78 (0.45, 1.33) *p* = 0.35	0.82 (0.5, 1.36) *p* = 0.45	0.79 (0.57, 1.11) *p* = 0.176	1.15 (0.72, 1.84) *p* = 0.55	1.03 (0.91, 1.16) *p* = 0.67	1.3 (0.54, 3.1) *p* = 0.56	1.03 (0.91, 1.16) *p* = 0.67
Model 3: As 2 + adjusted for gender	0.72 (0.36, 1.44) *p* = 0.35	0.77 (0.45, 1.33) *p* = 0.34	0.82 (0.5, 1.36) *p* = 0.44	0.79 (0.57, 1.11) *p* = 0.171	1.15 (0.72, 1.84) *p* = 0.55	1.03 (0.91, 1.16) *p* =0.67	1.29 (0.54, 3.1) *p* = 0.56	1.03 (0.91, 1.16) *p* = 0.67
Model 4: As 3 + adjusted for SES	0.71 (0.36, 1.44) *p* = 0.34	0.77 (0.45, 1.32) *p* = 0.34	0.82 (0.5, 1.36) *p* = 0.44	0.79 (0.56, 1.1) *p* = 0.168	1.15 (0.72, 1.83) *p* = 0.56	1.03 (0.91, 1.16) *p* = 0.68	1.29 (0.54, 3.09) *p* = 0.57	1.03 (0.91, 1.16) *p* = 0.68
Model 5: As 4 + adjusted for nature connectedness	0.75 (0.37, 1.51) *p* = 0.41	0.79 (0.46, 1.35) *p* = 0.38	0.84 (0.51, 1.39) *p* = 0.49	0.80 (0.57, 1.12) *p* = 0.19	1.15 (0.72, 1.84) *p* = 0.55	1.03 (0.91, 1.17) *p* = 0.62	1.25 (0.52, 3.0) *p* = 0.62	1.03 (0.91, 1.17) *p* = 0.62
Model 6: As 5 + living/work situation	0.77 (0.38, 1.56) *p* = 0.46	0.81 (0.47, 1.39) *p* = 0.41	0.86 (0.52, 1.43) *p* = 0.55	0.81 (0.58, 1.14) *p* = 0.21	1.04 (0.99, 1.1) *p* = 0.54	1.03 (0.91, 1.17) *p* = 0.61	1.27 (0.53, 3.07) *p* = 0.54	1.03 (0.91, 1.17) *p* = 0.61
Model 7: As 6 + level of education	0.78 (0.39, 1.54) *p* = 0.47	1.03 (0.86, 1.23) *p* = 0.75	0.87 (0.53, 1.43) *p* = 0.58	0.82 (0.59, 1.14) *p* = 0.23	1.26 (0.8, 2.0) *p* = 0.32	1.02 (0.91, 1.15) *p* = 0.68	1.42 (0.6, 3.34) *p* = 0.42	1.02 (0.91, 1.15) *p* = 0.68

Pr = presence; Ab = abundance
Odds ratio and 95% CI reported
†*n = 933.*
§Adjusted by index of multiple deprivation (IMD) quintiles
¶Based on nature relatedness-6 scale (NR-6)

Table S2. Breakdown of different OS green space typologies.

Types of Greenspace
Playing field
Other sports facility
Play space
Cemetery
Allotment or community garden
Religious grounds
Public park or garden
Bowling green
Tennis court
Golf course
Public park
Sports field
Grassland/scrub

Strategies used to reach participants

1. We used non-random sampling methods
2. We posted a link to our SmartSurvey questionnaire (along with participant information sheet and informed consent information) on social media platforms including Twitter, LinkedIn, and Facebook. These posts reached the authors network and people from this network also help by 'Retweeting' or forwarding the details of the survey on to other people in their networks.
3. We also posted the survey on volunteer email groups via the University of Sheffield.
4. We also carried out a manual webscrape of community groups – predominantly in the UK. This approach involved searching for publicly available 'community group directories' using the Google Search engine and tagging different geographical boundaries (e.g., UK counties) on to the search query. Community group directories were then identified and the email addresses of the group managers/coordinators were acquired. The authors then sent an ethically-approved approach email along with the questionnaire link to these groups.
